# Antimalarial activity of primaquine operates via a two-step biochemical relay

**DOI:** 10.1038/s41467-019-11239-0

**Published:** 2019-07-19

**Authors:** Grazia Camarda, Piyaporn Jirawatcharadech, Richard S. Priestley, Ahmed Saif, Sandra March, Michael H. L. Wong, Suet Leung, Alex B. Miller, David A. Baker, Pietro Alano, Mark J. I. Paine, Sangeeta N. Bhatia, Paul M. O’Neill, Stephen A. Ward, Giancarlo A. Biagini

**Affiliations:** 1 Centre for Drugs and Diagnostics Research, Tropical Disease Biology Department, Liverpool School of Tropical Medicine, Liverpool, L3 5QA UK; 2 Health Sciences and Technology/Institute for Medical Engineering and Science, Massachusetts Institute of Technology, Cambridge, MA 02139 USA; 3 Department of Chemistry, University of Liverpool, Liverpool, L69 7ZD UK; 4 Faculty of Infectious and Tropical Diseases, London School of Hygiene & Tropical Medicine, London, WC1E 7HT UK; 5 Dipartimento di Malattie Infettive, Istituto Superiore di Sanità, Rome, 00161 Italy; 6 Vector Biology Department, Liverpool School of Tropical Medicine, Liverpool, L3 5QA UK; 7 Present Address: ARUK Oxford Drug Discovery Institute, University of Oxford, Oxford, OX3 7FZ UK; 8 Present Address: Clinical Laboratory sciences Department, College of Applied Medical Sciences, Najran University, Najran, 61441 Saudi Arabia

**Keywords:** Malaria, Pharmacodynamics, Antiparasitic agents

## Abstract

Primaquine (PQ) is an essential antimalarial drug but despite being developed over 70 years ago, its mode of action is unclear. Here, we demonstrate that hydroxylated-PQ metabolites (OH-PQm) are responsible for efficacy against liver and sexual transmission stages of *Plasmodium falciparum*. The antimalarial activity of PQ against liver stages depends on host CYP2D6 status, whilst OH-PQm display direct, CYP2D6-independent, activity. PQ requires hepatic metabolism to exert activity against gametocyte stages. OH-PQm exert modest antimalarial efficacy against parasite gametocytes; however, potency is enhanced ca.1000 fold in the presence of cytochrome P450 NADPH:oxidoreductase (CPR) from the liver and bone marrow. Enhancement of OH-PQm efficacy is due to the direct reduction of quinoneimine metabolites by CPR with the concomitant and excessive generation of H_2_O_2_, leading to parasite killing. This detailed understanding of the mechanism paves the way to rationally re-designed 8-aminoquinolines with improved pharmacological profiles.

## Abstract (web summary)

Primaquine (PQ) is a widely used anti-malaria drug, but its mechanism of action is unclear. Here, Camarda et al. show that PQ’s activity against liver and sexual *Plasmodium* stages depends on generation of hydroxylated-PQ metabolites (OH-PQm), which, undergoing further reactions, results in production of H_2_O_2_.

## Introduction

**Fig. 1:**
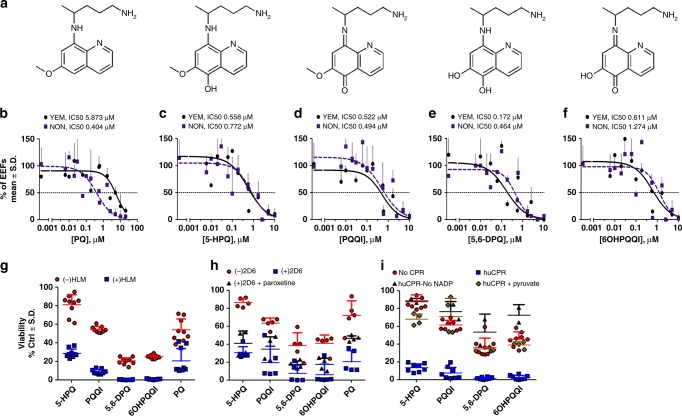
Structures of PQ and OH-PQm and their activity against *P. falciparum* liver stages and gametocytes. **a** Structures of compounds used in this study. **b**, **f** Dose dependent reduction in exoerythrocytic forms (EEFs) numbers at day 3.5 post infection with NF54 sporozoites in YEM (poor metaboliser, black line) and NON (extensive metaboliser, dashed blue line) hepatocyte lots. **b** Primaquine (PQ). **c** 5-hydroxy-primaquine (5-HPQ). **d** 5-quinoneimine (PQQI). **e** 5,6-dihydroxy-primaquine (5,6-DPQ). **f** 6-hydroxy-5-quinoneimine (6OHPQQI). Viability is expressed as mean percentage of vehicle control ± S.D. of two independent experiments performed in triplicates. For each drug, IC_50_ values are reported for the poor metaboliser, YEM, and extensive metaboliser, NON, hepatocyte lots. **g** Coupled in vitro metabolism-GC-LUC assay. PQ and PQ metabolites (30 μM) were reacted with (blue squares) or without (red circles human liver microsomes (HLM) prior to dilution to 10 μM (nominal parental compound concentration) in a GC-LUC assay with mature gametocytes. Viability was measured after 72 h and expressed as mean percentage of control (no drug) viability ± S.D. (*n* = 3, each in triplicate). **h** as in **g**, but with (blue squares) or without (red circles) CYP2D6 (*n* = 2, 5 total replicates). For paroxetine inhibition, CYP2D6 was pre-incubated with 10 μM paroxetine for 15 min prior to compounds (black triangles, *n* = 2, 4 total replicates); **i** as in **g**, but without (red circles) or with (blue squares) recombinant human CPR, or with huCPR in the presence of 10 mM sodium pyruvate (brown diamonds) with huCPR in the absence of NAPD^+^ (black triangles). NoNADP^+^ *n* = 1 in duplicate; all other huCPR conditions *n* = 4 in duplicate

Since its development in the 1950s by the US Army, an estimated 200 million people have been administered PQ^1^. Research into the elusive PQ mode of action has mainly focused on the identification of the biochemical basis for its side effect of hemolytic toxicity in patients with G6PD deficiency^1^. Earlier studies led to the identification of OH-PQm, generated from hepatic phase I metabolism^2^, as potential culprits for toxicity^3,4^. The most relevant species in this context include the 5-hydroxy (5-HPQ) and 5,6-dihydroxy (5,6-DPQ) primaquine metabolites (Fig. 1a). These species are unstable, undergoing spontaneous oxidation and producing the corresponding quinoneimine forms (Fig. 1a, PQQI, 5-quinoneimine, and 6OHPQQI, 6-hydroxy-5-quinoneimine) with concomitant generation of H_2_O_2_^5^; quinoneimine metabolites can then be reduced back to the hydroxylated form, leading to H_2_O_2_ accumulation. However, their reduction potential is likely to be very negative, limiting the pool of possible intracellular reductants with a sufficiently low reduction potential to donate electrons. In proof-of-concept studies using the strong reducing enzyme, ferredoxin-NADP reductase (FNR) from spinach (note the *E*_m,7_ known for the ferredoxin oxidised/reduced couple is ca. −430 mV), PQ quinoneimines were shown to be enzymatically reduced^5^—but despite intensive research efforts the exact enzyme(s) and metabolic pathway(s) involved in the process remain to be fully elucidated.

Similarly, the mechanism of action of primaquine against the malaria parasite is also largely unknown^6,7^. A link between drug efficacy and metabolism through CYP2D6 is supported by recent animal and clinical studies, including the association of CYP2D6 poor metaboliser phenotype status with primaquine failure in controlled human malaria infections with *P. vivax*^2,8^. The need for CYP2D6 metabolism to generate key OH-PQm^9,10^ supports the assumption that these metabolites contribute to drug efficacy; however, hitherto there has been no direct demonstration of the link between these metabolites and anti-parasitic activity. Any explanation of PQ action needs to address the exquisite selectivity against dormant and active liver stage parasites and the ability of very low drug doses and subsequent low systemic exposures to kill gametocyte stages of *P. falciparum* clinically^11^. This study attempts to reconcile these questions.

Here OH-PQm in their hydroxyquinoline or quinoneimine form have been synthesised and assessed for their activity against *P. falciparum* gametocytes and liver stages. By assessing parasite viability in the presence or absence of metabolic conversion we show that OH-PQm full cidal potential requires further enzymatic catalysis. We identify the CYP2D6 redox partner, cytochrome P450 NADPH:oxidoreductase (CPR), as required for OH-PQm to exert full gametocytocidal activity, independently of CYP2D6 status. We also show that OH-PQm activity against *P. falciparum* liver stage development is comparable or better than PQ and confirm a dispensable role for CYP2D6 activity. Finally, we substantiate the critical role of H_2_O_2_ production in anti-parasitic activity and propose a CPR-mediated biochemical model to account for PQ and OH-PQm mechanism of action. These data answer long-held questions of why PQ displays parasite stage-specific activity and why PQ displays transmission blocking activity at such low doses.

## Results

### Influence of CYP2D6 on PQ antimalarial liver-stage activity

To explore the activity of PQ and OH-PQm against *P. falciparum* liver stage development, we used an advanced in vitro model that consistently mimics liver physiology and supports complete maturation of *P. falciparum* extra-erythrocyte forms (EEFs) in the liver^12^. In this in vitro system, human primary hepatocytes are organised into colonies surrounded by supportive stromal cells (Micropatterned coculture, MPCC) and infected with *P. falciparum* (NF54 strain) sporozoites^12^. To evaluate the role of CYP2D6 in PQ and OH-PQm activity, we selected two hepatocyte lots, NON and YEM. YEM is a low metaboliser based on genotype analysis, it has a variation in the CYP2D6 allele (*4/*4); this was confirmed by incubation with the CYP2D6 probe debrisoquine^13^ and measuring the metabolite 4-hydroxidebrisoquine by mass spectrometry (MS) (Supplementary Fig. 1), confirming that the NON hepatocyte lot had twofold higher CYP2D6 activity than the YEM lot.

OH-PQm were synthesised and used alongside PQ to directly assess the inhibition of EEFs development in dose-response experiments in the two hepatocyte lots. As shown in Fig. 1b, PQ activity against EEFs development strictly depended on hepatocyte CYP2D6 status, with >14-fold increase in the IC_50_ measured in the YEM background (low metaboliser, PQ IC_50_ 5.87 μM) as compared to NON hepatocytes (extensive metaboliser PQ IC_50_ 0.40 μM). Conversely, all OH-PQm showed comparable IC_50_ values in the two hepatocyte lots (Fig. 1c–f), indicating that they act independently of CYP2D6 activity. It is interesting to note that all OH-PQm show similar potency against liver stages, comparable to that seen for PQ in the presence of extensive metaboliser hepatocytes. Taken together these results confirm that OH-PQm have direct killing activity against liver stages, supporting the hypothesis that they act downstream of CY2D6 activity.

### PQ gametocytocidal activity requires hepatic metabolism

We next explored the anti-gametocyte activity of OH-PQm and parental PQ through coupled in vitro metabolism-gametocytocidal assays. In these assays, we first incubated PQ and OH-PQm with human liver microsomes (HLM) to mimic bioactivation and then tested for gametocytocidal activity against a *P. falciparum* 3D7A transgenic strain, expressing luciferase specifically in the gametocyte stages^14^, in an improved gametocyte viability assay GC-LUC (gametocyte luciferase)^14^. As shown in Fig. 1g, PQ at 10 μM, as expected, has minimal activity in the absence of any metabolic conversion (red histograms, (−)HLM). When gametocytes were treated with OH-PQm, we observed little to moderate gametocytocidal activity, with only demethoxylated species, 5,6-DPQ and 6OHPQQI, able to reduce gametocyte viability to less than 50 % of solvent control (Fig. 1g, (−)HLM). After HLM metabolism (Fig. 1g, blue histograms, (+)HLM), PQ gametocytocidal activity significantly increased, confirming that metabolic transformation is also required for gametocyte killing activity. Interestingly, a significant increase in potency was also observed for all OH-PQm after microsomal incubation (*p* value < 0.001 for all pairs by Mann Whitney test, Supplementary Table 1). In both the presence and absence of metabolic conversion, 5,6-DPQ showed the highest gametocytocidal potency (Supplementary Table 2). These results confirm that PQ requires metabolic activation to elicit activity against both liver stage parasites and gametocytes; gametocytocidal activity of OH-PQm in the in vitro gametocyte setting is also shown to be HLM metabolism-dependent.

To gain further insight, we directly assessed the role of CYP2D6 in OH-PQm activity against gametocytes by performing coupled in vitro metabolism-GC-LUC assays using CYP2D6-expressing baculosomes (Fig. 1h). As with HLM, CYP2D6 treatment potentiated OH-PQm gametocytocidal activity, with *p* < 0.05 for 5,6-DPQ and 6OHPQQI, and *p* < 0.001 for all other compounds (Mann Whitney, Supplementary Table 3). However, OH-PQm are believed to be the terminal products of primaquine metabolism, so we further investigated these results by blocking CYP2D6 activity with the specific inhibitor paroxetine^15^ (Supplementary Fig. 2) prior to drug in vitro metabolism and GC-LUC assays. Interestingly, CYP2D6 inhibition did not significantly affect OH-PQm gametocytocidal activity (Fig. 1h, compare (+)2D6 and (+)2D6 + Paroxetine); conversely, PQ activity decreased (*p* < 0.05) as expected, reverting towards the levels observed in control samples without CYP2D6 (Mann Whitney, Supplementary Table 4). Overall, these results show that OH-PQm activity is greatly enhanced by baculosome metabolic component(s), however, the observed potentiation is independent of CYP2D6 activity.

These results prompted us to hypothesise that OH-PQm might be direct substrates of CPR, the CYPs redox partner required for electron transfer from NAPDH^16^, an intrinsic (and required) component of HLM and CYP2D6 baculosome preparations. We therefore measured initial reaction rates of human recombinant CPR (huCPR)^17^ with PQ and OH-PQm (Supplementary Fig. 5) and generated the apparent steady-state kinetics for each compound (Supplementary Table 5). These analyses revealed that OH-PQm are indeed CPR substrates; conversely, kinetic parameters showed parental PQ to be a poor CPR substrate, as predictable from its structure, and hence it was not included in the subsequent analysis. We then tested the gametocytocidal ability of OH-PQm following incubation with huCPR and observed that, akin to HLM and the baculosome treatment, in the presence of huCPR only, the gametocytocidal activity of all metabolites was significantly increased (Fig. 1i, *p* < 0.001 for all compounds, Mann Whitney, Supplementary Table 6). This effect was completely prevented when the assay was performed in the absence of NADP^+^, required for the production of the essential cofactor NADPH by the reaction components (see “Methods”) (Fig. 1i, compare (+)huCPR and (+)huCPR-No NADP^+^), confirming that huCPR catalysis mediates OH-PQm gametocytocidal activity. Cognisant of the putative role of H_2_O_2_-producing redox-active PQ metabolites in PQ haemolytic toxicity in G6PDH-deficient individuals^3,18^, we indirectly assessed the contribution of reactive oxygen species to the gametocytocidal activity of huCPR-treated PQ metabolites by using sodium pyruvate as a H_2_O_2_ scavenger^19^. As shown in Fig. 1i (compare huCPR and huCPR + pyruvate), the presence of pyruvate abrogated OH-PQm gametocytocidal activity (*p* < 0.01; Mann Whitney, Supplementary Table 7). These results establish the need for huCPR catalytic activity for OH-PQm mechanism of action and point to a direct role for huCPR as a redox cycler of OH-PQm.

### Redox cycling of PQ metabolites generates gametocytocidal H_2_O_2_

**Fig. 2:**
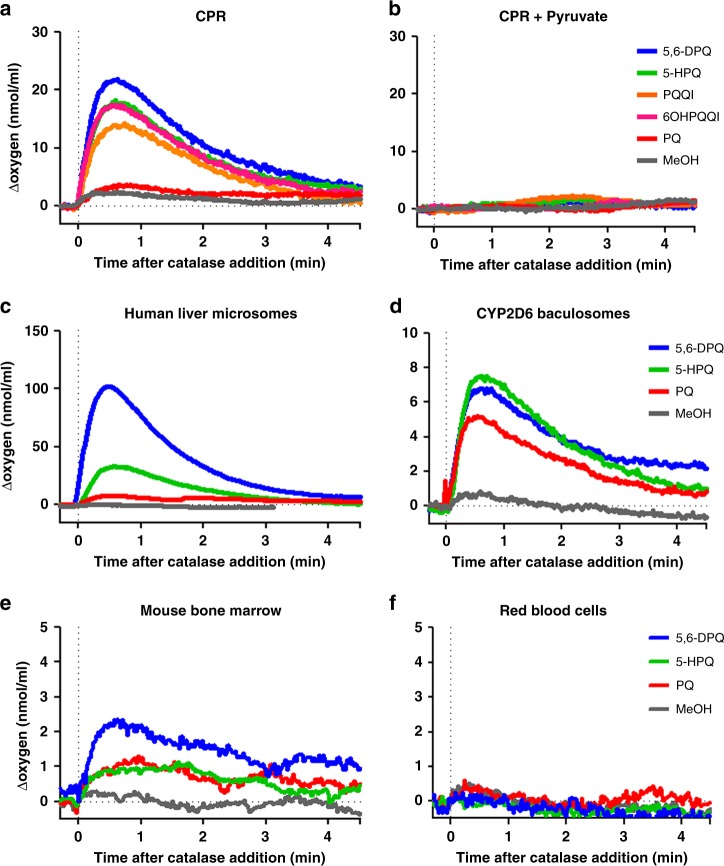
Metabolism-mediated generation of H_2_O_2_. H_2_O_2_ production was measured as catalase-mediated oxygen release after compound incubation (30 μM) with **a** human CPR, **b** human CPR + pyruvate, **c** human liver microsomes, **d** CYP2D6-expressing baculosomes, **e** mouse bone marrow extracts or **f** red blood cell extracts. The *x* axis was adjusted by defining the addition of catalase as *t* = 0, and the corresponding *y* axis value defined as 0 nmol mL^−1^. The average of two (PQ and 5,6-DPQ) and five (5-HPQ and MeOH) independent measurements are shown

To further confirm this observation, we directly measured H_2_O_2_ generation during drug incubation with human CPR (huCPR). To this end, O_2_ consumption during incubations was recorded using a closed O_2_ electrode and H_2_O_2_ was quantified via oxygen release after catalase addition. As shown in Fig. 2a, all metabolites, compared to methanol control, elicited H_2_O_2_ production, whereas negligible amounts of H_2_O_2_ were detected when PQ was used as a substrate. When sodium pyruvate was added to CPR reactions, H_2_O_2_ production was completely abolished (Fig. 2b). In addition, we measured H_2_O_2_ production in titration experiments with 5-HPQ as a representative OH-PQm (Supplementary Fig. 5) and observed that as low as 1 nM 5-HPQ is able to elicit H_2_O_2_ production. We confirmed H_2_O_2_ involvement in gametocyte killing by directly assessing its production upon HLM and CYP2D6 metabolism of PQ and 5-HPQ and 5,6-DPQ as representative metabolites (Fig. 2c, d and Supplementary Fig. 4 for representative full traces of oxygen content). Collectively, these data clearly demonstrate that huCPR is required for OH-PQm anti-gametocyte activity and that this process occurs through generation of H_2_O_2_, implying a redox cycling, CPR-mediated mechanism of parasite death directly attributable to the generation of H_2_O_2_.

### H_2_O_2_ production by PQ in bone marrow

We reasoned that spatial co-localisation of parasite and host-dependent metabolic activating system(s) could account for PQ stage-specificity. For EEFs this association is obvious with parasite residency within the hepatocyte; for gametocytes this is less obvious as circulating gametocytes are rarely in juxtaposition to a potential source of metabolism. However, *P. vivax* and *P. falciparum* gametocytes, including mature stages^20,21^, are sequestered/enriched in the bone marrow^22,23^, which is also endowed with significant P450-dependent metabolic capacity^24–26^. To address this possibility, the ability of PQ and OH-PQm to generate H_2_O_2_ in mouse bone marrow cell extracts was investigated. Results in Fig. 2e show that PQ and OH-PQm generated H_2_O_2_ when incubated with bone marrow crude cell extracts, whereas no H_2_O_2_ could be detected with equivalent amount of proteins from red blood cells (to represent the circulating parasite environment) (Fig. 2f). These results support the view that localised H_2_O_2_ generation at specific anatomical sites is responsible for stage-specific parasite killing by PQ metabolites.

### PQ-metabolite efficacy at pharmacologically-relevant doses

**Fig. 3:**
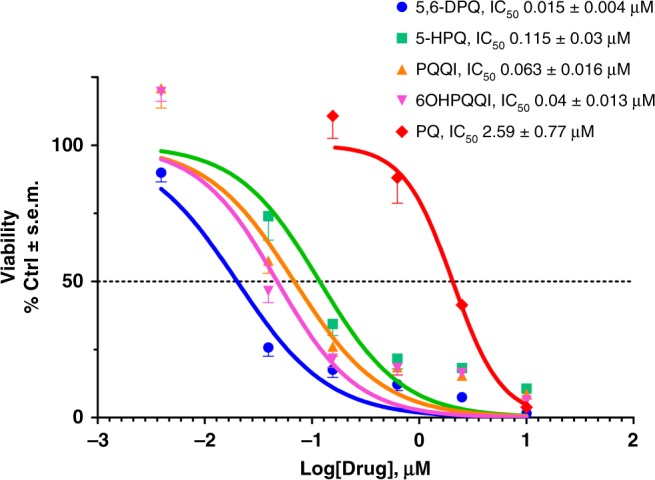
Dose-response viability of late stage gametocytes treated with PQ and OH-PQm upon reaction with human CPR. Compounds at concentrations ranging from 30 μM to 11.7 nM were incubated with human CPR and diluted 1:3 for GC-LUC assays (*n* = 2, each in triplicate). IC_50_s (±S.E.M.) were calculated from nonlinear regression (curve fit) in GraphPad Prism

Next, we determined gametocyte inhibition sensitivity profiles (IC_50_) of OH-PQm. Dose-response huCPR reactions were performed prior to GC-LUC assays. As shown in Fig. 3, OH-PQm are all active at pharmacologically-relevant nanomolar concentrations, with 5,6-DPQ being the most active with an IC_50_ of 15 ± 0.4 nM. Conversely, parental PQ shows an IC_50_ of 2.59 ± 0.77 μM, in strong agreement with the measured enzyme kinetics. These results support a catalytic mechanism of action for PQ metabolites via CPR-mediated redox cycling that is in line with expected drug metabolite exposure after clinically relevant PQ doses^27–29^.

## Discussion

**Fig. 4:**
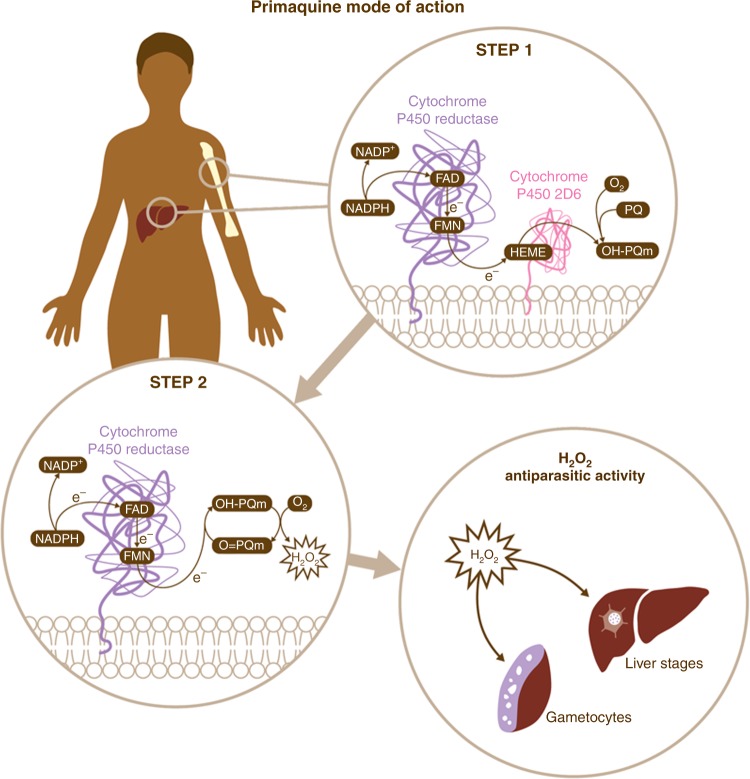
Schematic representation of primaquine mode of action. The results presented in this work support a two-step biochemical relay mechanism for PQ mode of action. In Step 1, PQ is converted into hydroxylated metabolites (OH-PQm) through the CPR/CYP2D6 metabolic complex. In Step 2, metabolites then undergo spontaneous oxidation to quinoneimines (O = PQm) with concomitant generation of H_2_O_2_. Human CPR then receives two electrons from NADPH and orchestrates one-electron transfers via FAD/FMN cofactors to quinoneinimes, thus reducing them back to the hydroxyl forms and perpetuating a catalytic cycle which brings about H_2_O_2_ accumulation at sites of metabolic transformation (liver, bone marrow and possibly others). *Plasmodium* parasites present at these locations are then killed by H_2_O_2_ action

Our results unveil a two-step biochemical relay underlying PQ mode of action relying on CYP2D6 and its redox partner CPR (Fig. 4). PQ oxidation principally by CYP2D6 generates hydroxyl-metabolites whose oxidation to quinoneimine generates H_2_O_2_. Quinoneimines in turn are substrates for CPR reducing activity, thus leading to H_2_O_2_ accumulation which can exert anti-parasitic activity directly through oxidation of protein sulphydryl groups and damage of Fe- and FeS-containing proteins or through the generation of superoxide and hydroxyl radicals. The intrinsic instability of parent hydroxyl-metabolites prevents identification of all the relevant chemical species in the process. However, it is important to emphasise that our results show that quinoneimine metabolites, considered as stable degradation products or markers for the unstable hydroxylated forms^28,29^, are also substrates for redox cycling activities involved in anti-parasitic action. Direct demonstration of CYP2D6-dependent PQ antimalarial activity against *P. falciparum* liver stages supports clinically-led hypotheses for the essentiality of CYP2D6 for PQ activity^8^.

Significantly, our data also shows that OH-PQm exert antimalarial activity against liver parasite stages independently of CYP2D6 (Fig. 2). This finding has significant implications for the design of improved 8-aminoquinolines which retain antimalarial activity but which importantly would not be determinately affected by the CYP2D6 status of the patient.

The described biochemical relay accounts for a unique mode of action (MoA) involving two host enzymes and has implications not only for bioactivation but also for pharmacodynamics. The elucidation of a catalytic (e.g. 1 nM) MoA for OH-PQm (generating μM levels of H_2_O_2_, Supplementary Fig. 5) explains how single low-dose PQ, down to 0.25 mg/kg, retains transmission blocking ability in clinical settings^30^; importantly, this treatment is also well tolerated in G6PD deficient patients^30^. The emphasis on a host-mediated MoA, not directly involving any parasite targets that are subject to selective pressure, may also provide an explanation for the apparent lack of resistance to PQ in the field; in fact, treatment failure so far has only been linked to CYP2D6 polymorphisms.

These results do not exclude a role for other human host strong reducing enzymes in mediating PQ antimalarial activity. However, we propose that the tissue-specific juxtaposition of CYP2D6 and CPR, in the liver and bone marrow^24–26^, exquisitely explains the susceptibility of liver stages, including hypnozoites, of *P. falciparum* gametocytes, and of *P. vivax* gametocytes and asexual stages, the latter recently shown to be enriched in the bone marrow^23^. Moreover, the results presented accurately mirror the clinical efficacy data in that PQ appears to be more active against gametocytes than liver stages. This observation supports the validity of the in vitro models used here as proxies for in vivo conditions.

Overall, these data provide a framework that will make it possible to test the potential of rationally re-designing PQ analogues that can be activated even against a backdrop of CYP2D6 poor metaboliser status. Furthermore, armed with this knowledge, it will be possible to test if antimalarial activity can ever be divorced from haemotoxicity.

## Methods

### Reagents

All chemical reagents were from Sigma-Aldrich unless otherwise specified.

### Chemical syntheses

Reactions that were air and moisture sensitive were performed under a nitrogen or argon atmosphere. This was achieved with oven dried or flame dried glassware sealed with a rubber septum. Dry nitrogen gas was introduced via a manifold or balloon.

Reactions were stirred using Teflon-coated magnetic stir bars. Organic solutions were concentrated using a Büchi rotary evaporator with a diaphragm vacuum pump. Anhydrous solutions and sensitive liquids were transferred via syringe.

All reagents were purchased from Sigma Aldrich or Alfa Aesar and were used without purification unless otherwise indicated. 5,6-dimethoxy-8-nitroquinoline was obtained from WuXi AppTec.

^1^H NMR spectra were measured on a Brucker AMX400 (400 MHz) nuclear magnetic resonance spectrometer. Solvents are indicated in the text. The data for ^1^H NMR spectra are reported as follows: chemical shifts were described in parts per million (δ, ppm) downwards from an internal reference of trimethylsilane. ^13^C NMR spectra were measured on a Brucker AMX400 (100 MHz) and are reported in terms of chemical shift (δ, ppm) relative to residual solvent peak. MS and High resolution mass spectrometry (HRMS) were recorded on a VG analytical 7070E machine, Frisons TRIO spectrometers or Agilent QTOF 7200 using chemical ionisation (CI) or electron ionisation (EI). Micromass LCT mass spectrometer used electron spray ionisation (ESI).

The synthesis of metabolites was based on modifications of literature procedures (Supplementary Fig. 6)^31,32^ as described below.

For the synthesis of 5,6-dimethoxyquinolin-8-amine^31^ 5,6-dimethoxy-8-nitroquinoline (2 g, 8.54 mmol) was dissolved in Tetrahydrofuran (THF, 20 mL). A solution of sodium hypophosphite (6 g, 68.32 mmol) in 10 mL water was added to the reaction. The flask was purged with nitrogen. 10% palladium on carbon (0.7 g) was added to the reaction mixture and purged with nitrogen. The reaction was allowed to stir vigorously for 10 min. A celite pad was prepared and the reaction mixture was filtered through the pad of celite and washed through with 20 mL chloroform. Sodium hydroxide (2 M, 50 mL) was added to the filtrate. The filtrate was extracted with chloroform (3 × 100 mL) and the combined organic extracts were washed with water (2 × 50 mL), dried over magnesium sulphate and filtered. The chloroform was removed under reduced pressure to obtain the pure product (1.72 g, 98.5%) as a red-orange solid; *R*_f_ = 0.15, 30 % ethyl acetate in hexane; ^1^H NMR (400 MHz, CDCl_3_) δ 8.62 (dd, *J* = 4.1, 1.6 Hz, 1 H), 8.33 (dd, *J* = 8.5, 1.6 Hz, 1 H), 7.35 (dd, *J* = 8.5, 4.1 Hz, 1 H), 6.73 (s, 1 H), 4.87 (s, 2 H), 3.96 (s, 3 H) and 3.89 (s, 3 H); ^13^C NMR (100 MHz, CDCl_3_) δ 149.6, 145.6, 141.5, 133.8, 133.2, 129.9, 124.5, 121.6, 99.1, 61.6 and 56.8; IR *ν*_max_ (neat)/cm^−1^ 3443, 3337 (NH), 3192 (CH) and 1623 (C=C); HRMS calculated for C_11_H_12_N_2_O_2_ [M + H]^+^ 205.0972 found 205.0977.

For the synthesis of 4-bromo-1-phthalimidopentane, potassium phthalimide (10 g, 53.99 mmol) was dissolved in acetone. 1,4-Dibromopentane (9.57 mL, 70.19 mmol) was added to the mixture which was heated to reflux for 24 h. The reaction was cooled and filtered. The acetone was removed under reduced pressure and the crude product was purified via flash chromatography resulting in (14.62 g 91%) a clear light yellow oil. *R*_f_ = 0.43, 20% ethyl acetate in hexane; ^1^H NMR (400 MHz, CDCl_3_) δ 7.88–7.81 (m, 2 H), 7.76–7.70 (m, 2 H), 4.22–4.10 (m, 1 H), 3.72 (dd, *J* = 8.5, 4.7 Hz, 2 H), 2.02–1.74 (m, 4 H) and 1.70 (d, *J* = 6.7 Hz, 3 H); ^13^C NMR (100 MHz, CDCl_3_) δ 168.4, 134.1, 132.1, 123.3, 50.6, 38.1, 37.2, 27.1 and 26.5; HRMS Calculated for C_13_H_14_BrNO_2_ [M + H]^+^ 296.0281 found 296.0280. 5,6-Dimethoxy-8-aminoquinoline (500 mg, 2.45 mmol) was added to a dry 50 mL two neck flask along with a dry magnetic bar. A dry condenser and equilibrating dropping funnel were attached to the flask and the second neck was sealed with a rubber septum. The system was purged with dry argon and sealed with a balloon attached to the dropping funnel. 1-Phthalimido-4-bromopentane (942.6 mg, 3.18 mmol) was added directly to the reaction via syringe and the reaction was then heated to 150 °C to produce a dark paste. Triethylamine (444 µL, 3.18 mmol) was added to the reaction via the dropping funnel over a 1.5 h period. The reaction was left to stir for a further 1.5 h. Additional 1-phthalimido-4-bromopentane (1.23 g, 4.16 mmol) was added directly to the reaction via syringe. Triethylamine (290.3 µL, 2.08 mmol) was added to the reaction via the dropping funnel over 30 min. The reaction was left to stir for a further 2 h. 1-Phthalimido-4-bromopentane (235.7 mg, 795.7 µmol) was added to the reaction directly via syringe. Triethylamine (122.9 µL, 881.4 µmol) was added to the reaction via the dropping funnel over 30 min. The reaction was left to stir for a further 2 h and monitored by thin layer chromatography (TLC). The reaction was cooled, diluted with acetone and filtered. The acetone was removed, resulting in a dark oil which was dissolved in chloroform (100 mL). The organic layer was washed with water (3 × 50 mL), dried over magnesium sulphate and filtered. The crude product was pre-absorbed onto silica and purified via flash chromatography to obtain the product (790.5 mg, 77%) as a yellow oil; *R*_f_ = 0.3, 30% Ethyl Acetate in Hexane; ^1^H NMR (400 MHz, CDCl_3_) δ 8.53 (dd, *J* = 4.1, 1.6 Hz, 1 H), 8.27 (dd, *J* = 8.5, 1.6 Hz, 1 H), 7.84–7.78 (m, 2 H), 7.72–7.66 (m, 2 H), 7.33 (dd, *J* = 8.5, 4.1 Hz, 1 H), 6.41 (s, 1 H), 5.89 (d, *J* = 6.4 Hz, 1 H), 3.99 (s, 3 H), 3.86 (s, 3 H), 3.74 (t, *J* = 7.1 Hz, 2 H), 3.71–3.63 (m, 1 H), 1.99–1.62 (m, 4 H) and 1.30 (d, *J* = 6.3 Hz, 3 H); ^13^C NMR (100 MHz, CDCl_3_) δ 168.6, 150.0, 144.8, 141.9, 134.0, 133.7, 132.2, 131.3, 129.8, 124.6, 123.3, 121.7, 94.3, 61.6, 57.0, 48.1, 38.1, 34.1, 25.5 and 20.8; IR *ν*_max_ (neat)/cm^−1^ 3390 (NH), 2964, 2936 (CH) and 1707 (C=O); HRMS Calculated for C_24_H_25_N_3_O_4_ [M + H]^+^ 420.1923 found 420.1922.

For the synthesis of 5,6 dimethoxy primaquine^31^, 5,6 dimethoxy-phthaloyl primaquine (790.5 mg, 1.88 mmol) was dissolved in 25 ml of ethanol in a 50 mL round bottom, a condenser was attached and the system was purged with nitrogen. Sixty-five percent hydrazine monohydrate (465.5 µL, 6.22 mmol) was added to the reaction and refluxed (at 100 °C) for 6 h. A solid precipitate was observed. The reaction was cooled and filtered. The ethanol was removed and 30% potassium hydroxide (100 mL) was added to the residue. The mixture was extracted with diethyl ether (3 × 50 mL). The combined organic layers were washed with water (50 mL), dried over magnesium sulphate and filtered. A solution of 89% phosphoric acid (187 µL, 2.73 mmol) in ethanol (5 mL) was added drop wise to the ether solution. A red/orange precipitate was observed. The solvent was removed under vacuo and the precipitate was recrystallised with ethanol to obtain an orange/red solid (501 mg, 92%); ^1^H NMR (400 MHz, DMSO) δ 8.57 (dd, *J* = 4.1, 1.5 Hz, 1 H), 8.23 (dd, *J* = 8.5, 1.5 Hz, 1 H), 7.47 (dd, *J* = 8.5, 4.1 Hz, 1 H), 6.55 (s, 1 H), 6.02 (s, 2 H), 3.94 (s, 3 H), 3.75 (s, 3 H), 3.80–3.71 (m, 1 H), 2.80 (t, *J* = 6.3 Hz, 2 H), 1.79 –1.55 (m, 4 H) and 1.23 (d, *J* = 6.2 Hz, 3 H); ^13^C NMR (100 MHz, DMSO) δ 149.8, 144.6, 141.5, 132.6, 130.0, 129.2, 123.8, 122.0, 94.0, 60.9, 56.5, 46.9, 38.9, 33.1, 24.1 and 20.4; HRMS Calculated for C_24_H_25_N_3_O_4_ [M + H]^+^ 420.1923 found 420.1922.

Two general procedures were used for demethylation, as indicated. The general procedure 1 was based on a modified procedure of Allahyari et al.^32^. Ten milligram of 5,6 dimethoxy primaquine was dissolved in 1 mL of 48% hydrogen bromide solution (ca 6 M) and sealed under argon in a sealed tube. The reaction was heated to 120 °C on a pre-heated mantle for 20 mins. Under a flow of nitrogen, 5 mL of water was added and the product(s) were purified via HPLC. For the general procedure 2, 10 mg of 5,6 dimethoxy primaquine was dissolved in 1 mL of 48% hydrogen bromide solution and the solution was allowed to stir for 6 h. Excess reagent was removed under vacuum to give a brown solid that was stored under nitrogen.

HPLC conditions were as follows: a Phenomenex Jupiter Proteo 90 A column, 250 × 10 mm, 10 Micron was used for purification using a gradient system based on the following conditions; Initial (time 0) solvent mix 5% acetonitrile, 95% 0.05% trifluoracetic acid (TFA) in water; 20 mins, 25% acetonitrile, 75% 0.05% TFA in water; 20.10 min 5% acetonitrile, 95% 0.05% TFA in water; flow rate was 5 ml min^−1^.

All samples were dried by blowing off the solvent under a flow of nitrogen 5,6-dihydroxyprimaquine (5,6-DPQ) was prepared according to general procedure 2; this compound was very unstable rapidly oxidising to 5-hydroxy quinoneimine (5-HPQ) in solutions exposed to air. (MS for C_14_H_19_N_3_O_2_ [M + H]^ + ^found 262.33). The metabolite was stored under nitrogen in a sealed tube.

For the synthesis of 5-hydroxy primaquine (5-HPQ) general procedure 1 along with HPLC purification and drying was used (see HPLC conditions above). 5-Hydroxy 6-Methoxy Primaquine = 10.06 min. The 5-hydroxy 6-methoxy primaquine was very unstable as it readily oxidises to the quinoneimine form. LCMS has shown that this is present within the reaction mixture, however, upon isolation and re-evaluation using the HPLC conditions, the retention time at 10.06 min corresponding to the product is no longer observed. Once the quinoneimine has formed from 5-OH primaquine, it is readily converted to the 6-hydroxy form (6OHPQQI; Rt = 12.55 mins) by a demethylation reaction (through reaction with water).

For the synthesis of Primaquine quinone-imine (PQQI), general procedure 1 provides the -hydroxy primaquine which can be allowed to oxidise to the quinoneimine in aqueous solution. HPLC purification and drying (see above) were used for isolation. The quinoneimine was purified with two HPLC purification runs. Retention time: = 7.76 min ^1^H NMR (500 MHz, D_2_O) δ 9.03 (d, *J* = 3.6 Hz, 1 H), 8.59 (d, *J* = 6.6 Hz, 1 H), 7.96 (dd, *J* = 8.0, 4.8 Hz, 1 H), 6.99 (s, 1 H), 4.22 (s, 3 H), 3.07 (t, *J* = 7.6 Hz, 3 H), 2.11–1.95 (m, 3 H), 1.88–1.72 (m, 3 H) and 1.58 (d, *J* = 6.5 Hz, 3 H); MS for C_15_H_19_N_3_O_2_ [M + H]^+^ found 274.38.

For the synthesis of 6-hydroxy primaquine quinone imine (6OHPQQI), general procedure 2 along with HPLC purification and drying (see section 1.1.3) was used. This compound could also be produced by reaction of the 6-methoxy quinoneimine in aqueous solution followed by HPLC purification. (See analysis below) Retention time: = 12.55 min. ^1^H NMR (500 MHz, D_2_O) δ 8.86 (dd, *J* = 4.9, 1.7 Hz, 1 H), 8.40 (dd, *J* = 7.9, 1.7 Hz, 1 H), 7.75 (dd, *J* = 7.9, 4.9 Hz, 1 H), 6.12 (s, 1 H), 4.11 (dd, *J* = 13.1, 6.7 Hz, 1 H), 3.05 (t, *J* = 7.4 Hz, 2 H), 1.92–1.74 (m, 4 H) and 1.42 (d, *J* = 6.5 Hz, 3 H); MS for C_14_H_17_N_3_O_2_ [M + H]^+^ found 260.35.

### In vitro metabolism and redox cycling reactions

Human liver microsomes (HLM, BD Biosciences) and CYP2D6-expressing baculosomes from the Vivid CYP450 kit (Life Technologies) were used for compound metabolic conversions as per manufacturer’s instructions with minor modifications. For HLM, compounds (30 μM final concentration) were incubated in the presence of NAPDH Regeneration System Solution A and B (BD Biosciences) at 37 °C for 2 h in a total volume of 100 μl of phosphate reaction buffer. For CYP2D6 baculosomes reaction, a 2× master mix (50 μl) was prepared containing reaction buffer, baculosomes, regeneration system and NADP^+^ (all from Life technologies). Compounds (30 μM final concentration) were prepared in 40 μl reaction buffer containing NADP^+^ as per kit protocol. The total volume was brought to 100 μl with reaction buffer and reactions incubated at 37 °C for 2 h. For CYP2D6 inhibition, we first determined paroxetine inhibitory profile using Vivid CYP2D6 Blue kit (Life Technologies) according to manufacturer’s instructions. For gametocytocidal assays, master mixes without compounds were pre-incubated with paroxetine (10 μM) for 15 min at 37 °C before addition of compounds and further incubation 37 °C for 2 h. Recombinant huCPR^17^ was used for in vitro redox cycling of compounds. Reactions were performed as described above with minor modifications. A master mix without baculosomes was prepared as above; huCPR were added in 10 μl of reaction buffer (200 nM final concentration) and reactions started by adding 40 μl of compound/NADP^+^ mix. For H_2_O_2_ scavenging, sodium pyruvate (10 mM) was used, without pre-incubation. All experiments contained control reactions with solvent (MeOH:water 50:50) only. After incubations, reaction mixes were spun down, supernatants collected, diluted 2:3 and seeded into 96-well plates for GC-LUC assay.

### Parasite culture, drug treatments and gametocyte luciferase assay (GC-LUC)

A *P. falciparum* 3D7A^33^ transgenic derivative *3D7elo1-pfs16*-CBG99 was used^14,34^, specifically expressing the CBG99 luciferase in gametocytes. Parasites were cultured^35^ in human 0 + erythrocytes at 5% haematocrit under 5% CO_2_, 2% O_2_, 93% N_2_. Cultures were grown in complete medium (CM) containing RPMI 1640 medium (Gibco) supplemented with 25 mM Hepes (VWR), 50 μg mL^−1^ hypoxanthine, 0.25 mM NaHCO_3_, 50 μg mL^−1^ gentamicin sulfate, and 10% pooled heat inactivated AB + human serum. Gametocyte viability was evaluated by the GC-LUC assay^14,34^. Parasites were quickly harvested in 2× CM before addition to 96-well plates containing control, CYP2D6- or huCPR-treated compounds (10 μM parental compound final concentration) in aqueous solution and incubatied at 37 °C for 72 h. Drug-treated gametocytes were then transferred to 96-well white plate; D-Luciferin, 1 mM in 0.1 M citrate buffer pH 5.5 (Promega), was added in a 1:1 ratio and luminescence measurements were recorded after 10 min on a FLUOstar Omega plate reader (BMG Labtech). Viability was expressed as % viability as compared to solvent treated controls.

### Liver stages assay

Micropatterned hepatocyte-fibroblast co-cultures were established as previously described^12,36^. Briefly, soft lithography techniques were used to pattern rat tail type I collagen (Corning) into 500 µm diameter islands on the surface of glass bottom 96-well plates. Cryopreserved primary human hepatocytes (Bioreclamation IVT) were thawed and pelleted through centrifugation at 100 g for 6 min, assessed for viability using trypan blue exclusion (70–90% viability) and 10,000 hepatocytes were seeded onto the collagen islands in serum-free DMEM (Dulbecco’s Modified Eagle’s medium) with 1% Penstrep. Two to three hours later, cells were washed with DMEM containing 1% Penstrep, and media was replaced with hepatocyte culture media. The following day, each well was infected with 70,000 fresh *P. falciparum* sporozoites. Three hours later, cultures were washed with DMEM containing 3% Penstrep and 0.1% Fungizone, and 7000 3T3 J2 mouse fibroblasts were added to establish the co-culture. Drug was administered during daily media changes for 3 days. The impact on hepatocyte infection was measured by enumeration of exoerythrocytic forms on day 3.5 post infection, through staining for *Pf*HSP70 and visualisation with a Nikon Eclipse Ti fluorescent microscope.

### Determination of oxygen consumption and H_2_O_2_ production

Oxygen consumption and H_2_O_2_ production measurements were performed using the Oxytherm system and O_2_ View software package v.2.06 (Hansatech Instruments Ltd). Compounds ability to generate hydrogen peroxide after in vitro metabolism or huCPR reaction, in the presence of regeneration system or 100 μM NADPH as indicated, was assessed indirectly by measuring catalase-mediated oxygen release. Once the kinetic trace for oxygen concentration within the mixture had reached a plateau for at least 3 min, catalase (from bovine liver, prepared in 50 mM potassium phosphate buffer, pH 7.0; final assay concentration 10 µg ml^−1^) was added and data recorded for a further 6 min. Oxygen concentration was recorded as nmol mL^−1^. To allow for easier comparison of individual traces, the *x* axis was adjusted by defining the addition of catalase as *t* = 0, and the corresponding *y* axis value defined as 0 nmol mL^−1^.

For bone marrow experiments, femurs were dissected from mice following schedule 1 procedure. The schedule 1 procedure was undertaken with local (LSTM and UoL Animal Welfare Ethics Review Boards) and national (Home Office licence) authorisation. Bone marrow cells were flushed out with cold Ringer’s solution pH 7.4 (125 mM NaCl, 1.5 mM CaCl_2_, 5 mM KCl, 0.8 mM Na_2_HPO_4_) using 2 ml syringe connect with a 25GA needle. The bone marrow cells were washed twice and resuspended with cold Ringer’s solution. Cells were lysed by sonication and the protein concentration of crude extracts measured. Oxygen measurements were performed at 37 ˚C in 0.4 ml samples containing Ringer’s solution pH 7.4, 8.5 mg ml^−1^ bone marrow extracts, 1X regeneration system (Thermo Scientific), 30 µM PQ metabolites, 30 µM NADP^+^. The assay mixture without NADP^+^ was pre-incubated in the Oxytherm’s chamber at 37˚C while recording oxygen content. Then, the reaction was initiated by addition of NADP^+^. After 30, 10 µl of 5 mg ml^−1^ catalase (prepared in 50 mM potassium phosphate buffer pH 7.0) was added to release O_2_ from H_2_O_2_. Experiments with red blood cells were performed as above with same amount of protein extracts.

### Steady-state kinetic measurements

Kinetic measurements were determined following Tsukamoto et al.^37^ and the assay conditions were optimised according to Döhr et al.^38^ with minor modifications. Briefly, huCPR kinetics of interaction with PQ and OH-PQm were measured as NADPH consumption via recording of change in absorbance at 340 nm on a microplate spectrophotometer (Thermo Electron Varioskan) at 25 °C. The activity assay of 0.3 ml contained 0.3 M potassium phosphate (pH 7.7), 25 nM huCPR, 100 µM NADPH, and various concentrations of primaquine derivatives (0–200 µM 5-HPQ, 0–100 µM 5,6-DPQ, 0–150 µM PQQI, 0–150 µM 6OHPQQI, or 0–1000 µM PQ). Reactions were initiated by adding NADPH to a final concentration of 100 µM after equilibrating the assay mixture with all other components at 25 °C for 2 min. Data were recorded every 5 s over 4 min. The Michaelis–Menten equation was used to determine *K*_m_ and *k*_cat_ values.

### Statistics

Statistical analyses were done using GraphPad Prism version 5.04 and version 7 software (GraphPad Software, San Diego, California, USA, www.graphpad.com). Significance was calculated by two-tailed Mann Whitney test. One-way or two-way ANOVA was used as appropriate. Extended statistics tables for each analysis are provided as Additional Information.

### Reporting summary

Further information on research design is available in the Nature Research Reporting Summary linked to this article.

## Supplementary information

Supplementary Information

Reporting Summary

Source Data

## Data Availability

The source data underlying Figs. 1a, 2a–d, 6d, h and 7c and Supplementary Figs. 1a and 5d are provided as a Source Data file.
