# Supplementary material for: Antimalarial activity of primaquine operates via a two-step biochemical relay
Source: Nat Commun. 2019 Jul 19;10:3226. doi: 10.1038/s41467-019-11239-0 (PMC6642103; doi:10.1038/s41467-019-11239-0)
Supplement: Supplementary file 1 — Supplementary Information [file 41467_2019_11239_MOESM1_ESM.pdf]

Supplementary Information

Antimalarial activity of primaquine operates via a two-step biochemical relay

Camarda *et al.*

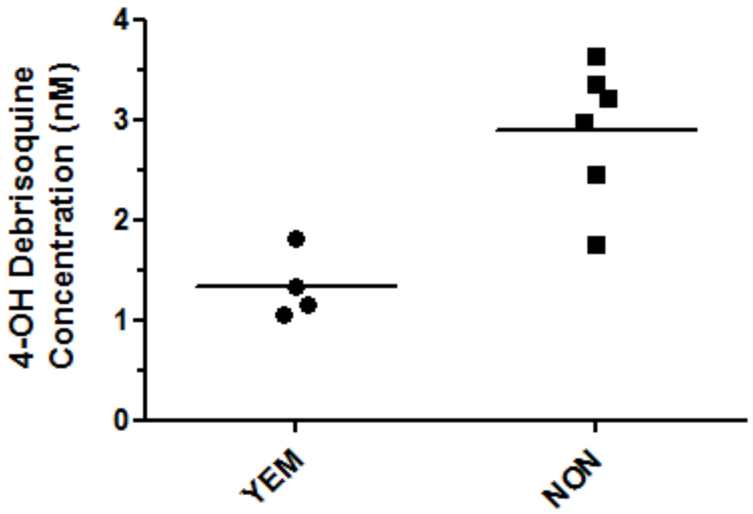

Supplementary Figure 1. CYP2D6 functional assay in YEM and NON hepatocyte lots. Debrisoquine, (100 μM) was added to hepatocyte cultures and collected after 1 h. The amount of 4-hydroxydebrisoquine was measured in the collected supernatant by LC/MS/MS. A single experiment was performed, with 4 and 6 replicates for YEM and NON lots, respectively.

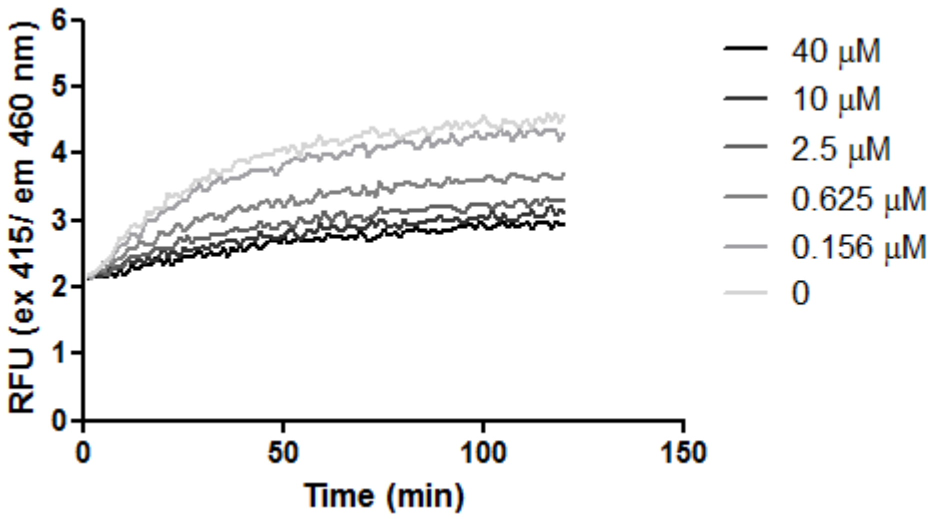

Supplementary Figure 2. Dose-response inhibition of CYP2D6 activity by paroxetine. CYP2D6 activity in the presence of different concentrations paroxetine was measured by recording the conversion of Vivid BOMCC substrate (Life Technologies) into a fluorescent product. Fluorescence was recorded using a microplate spectrophotometer (The Thermo Electron Varioskan). A single experiment was performed.

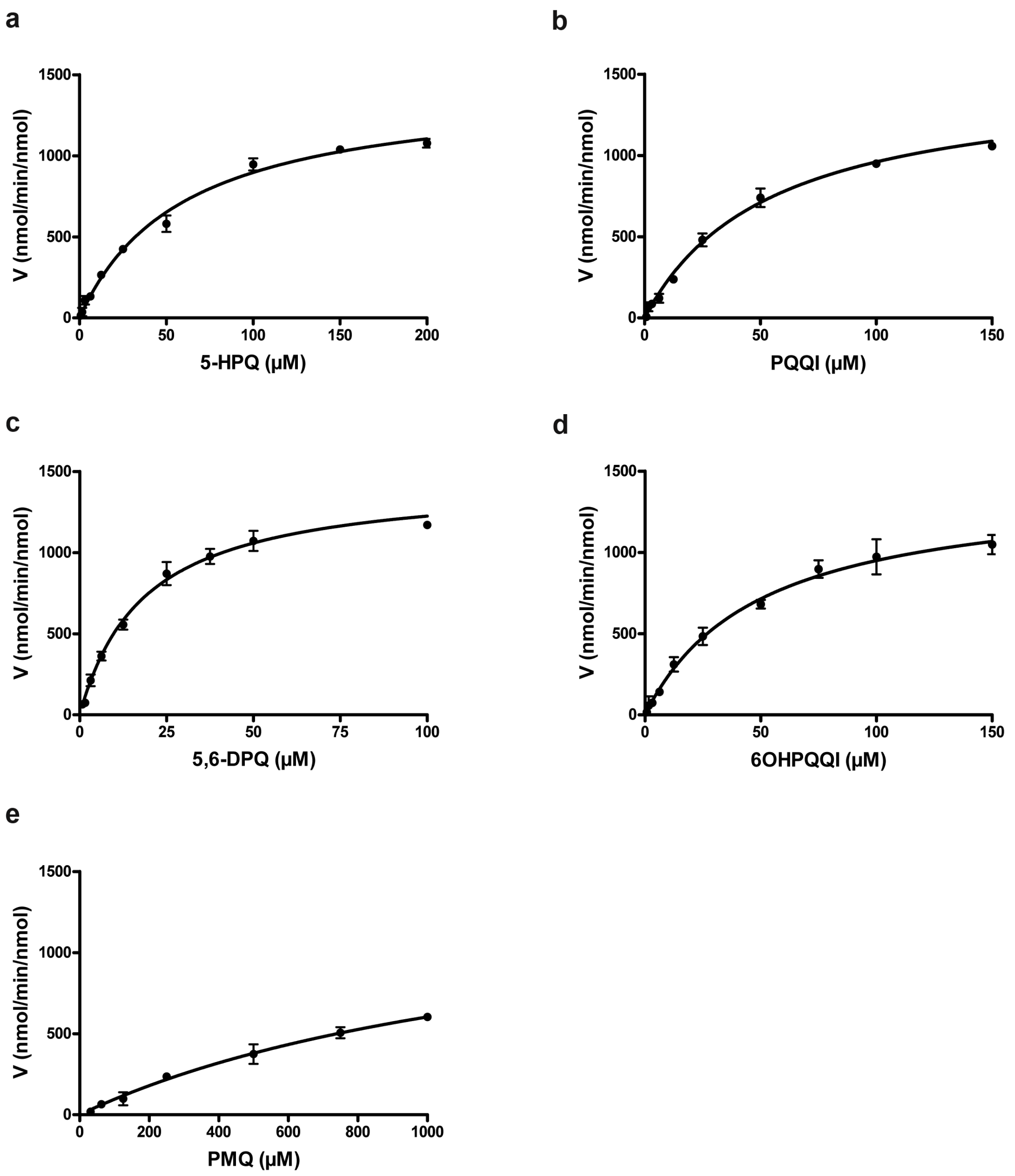

Supplementary Figure 3. Initial reaction rates for human CPR with different primaquine derivatives. NADPH consumption was measured with 25 nM human CPR and various primaquine metabolites concentrations. Initial reaction rates were obtained from four (PQQI, 6OHPQQI, PQ) or five (5-HPQ, 5,6-DPQ) determinations from two independent experimental assay sets. Solid lines represent fits of data to Michaelis–Menten equation using GraphPad Prism v7. **a**, 5-HPQ; **b**, PQQI; **c**, 5,6-DPQ; **d**, 6OHPQQI; **e**, PQ.

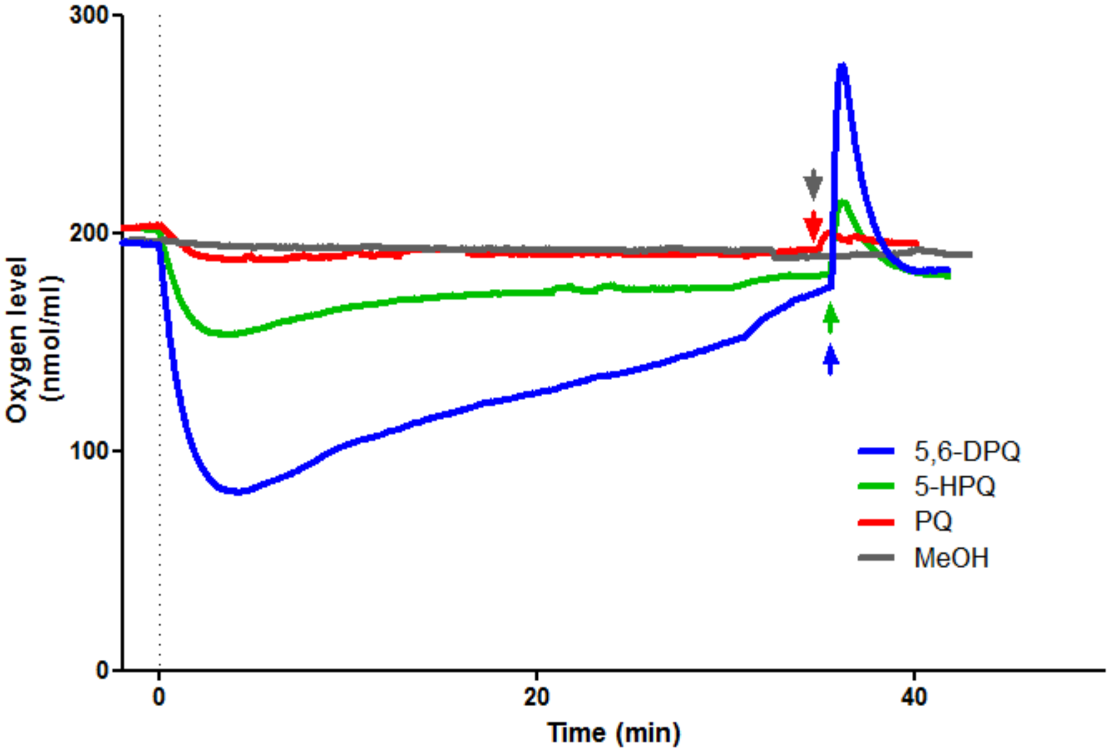

Supplementary Figure 4. Representative full trace of oxygen levels during liver microsomes metabolism of PQ and OH-PQm. Before compound additions, oxygen levels were recorded until stabilisation. PQ, OH-PQm and methanol as a control were added at time t=0. When oxygen levels approached the initial values, catalase was added (color coded arrows).

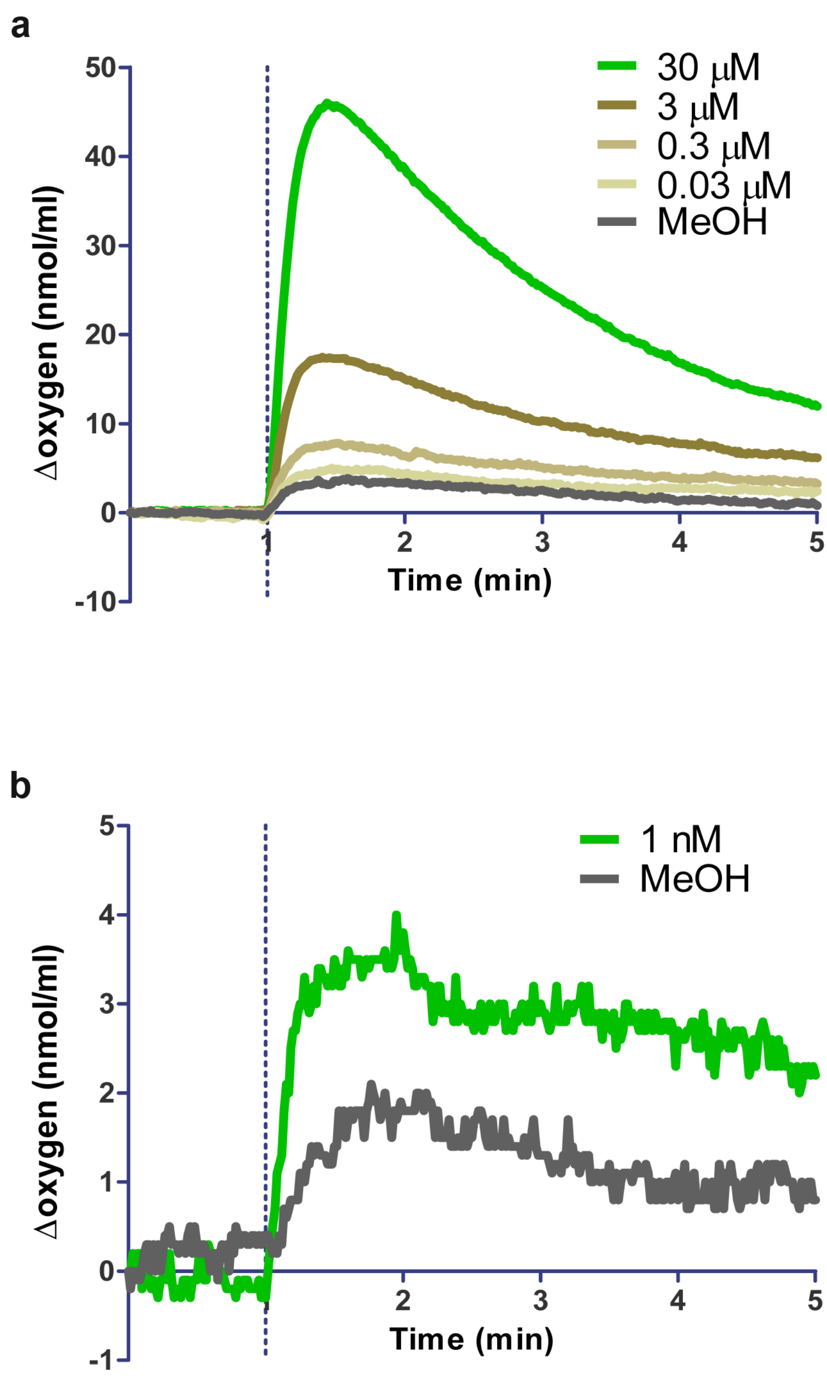

Supplementary Figure 5. H_2O_2 generation by nanomolar addition of 5-HPQ upon incubation with human CPR. **a,** H_2O_2 production was measured as catalase-mediated oxygen release after incubation (30 – 0.03 μM) with human CPR; **b,** 5-HPQ at 1 nM was incubated 4 h with human CPR before addition of catalase and oxygen release measurement. The *x* axis was adjusted by defining the addition of catalase as t=0, and the corresponding *y* axis value defined as 0 nmol mL^{-1}. The average of two independent experiments are shown.

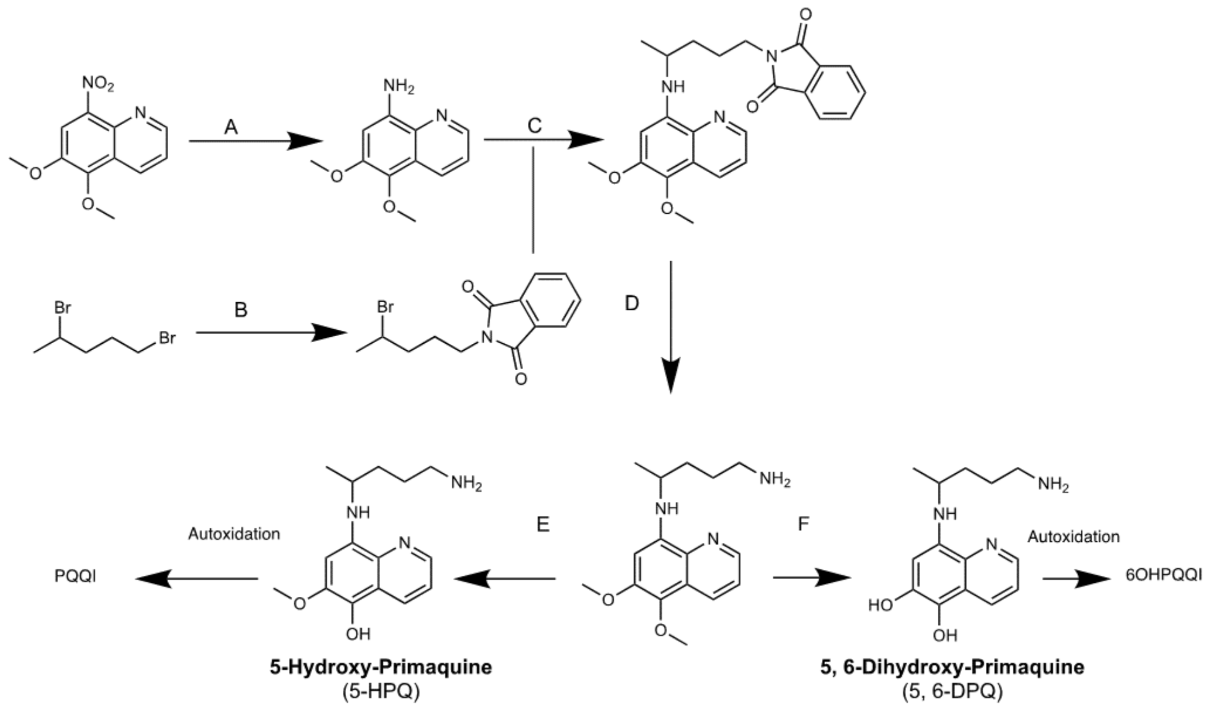

Supplementary Figure 6. Synthesis of Primaquine Metabolites (A) THF, Sodium hypophosphite in water, 10% Pd/C, N_2, 10 min, r.t, **99%**. (B) Potassium phthalimide, Acetone, Reflux, 24 h, **91%**; C (i) 1-phthalimido-4-bromopentane (1.3 eq), 150°C, Ar(g) (ii) NEt_3 (1.3 eq) added dropwise over 1.5 h. stir 1.5 h (iii) 1-phthalimido-4-bromopentane (1.7 eq) (iv) NEt_3 (0.85 eq) dropwise over 30 min, stir 2 h (v) 1-phthalimido-4-bromopentane (0.325 eq) (vi) NEt_3 (0.36 eq) dropwise over 30 min, stir 2 h, **77%**. (D) Hydrazine monohydrate (3.3 eq), Ethanol, reflux, 6 h, **97%**; (E) 48% HBr in water, Argon, Reflux at 120°C, 20 Min, **~79%** recovery very unstable (F) 48% HBr in water, Argon, 6 h.

Supplementary Table 1

Comparison of anti-gametocyte activity without and after metabolic conversion.

Table Analyzed	5-HPQ HLM	PQQI HLM	5,6-DPQ HLM	6OHPQQI HLM	PQ HLM
Column A	(-)HLM	(-)HLM	(-)HLM	(-)HLM	(-)HLM
vs	vs	vs	vs	vs	vs
Column B	(+)HLM	(+)HLM	(+)HLM	(+)HLM	(+)HLM
Mann Whitney test					
P value	< 0.0001	< 0.0001	0.0004	< 0.0001	< 0.0001
Exact or approximate P value?	Exact	Exact	Gaussian Approx	Exact	Exact
P value summary	****	****	***	****	****
Are medians signif. different? (P < 0.05)	Yes	Yes	Yes	Yes	Yes
One- or two-tailed P value?	Two-tailed	Two-tailed	Two-tailed	Two-tailed	Two-tailed
Sum of ranks in column A,B	126 , 45	126 , 45	126 , 45	126 , 45	126 , 45
Mann-Whitney U	0	0	0	0	0

Supplementary Table 2

GC-LUC assay survival (mean % of vehicle control).

	(-)HLM	(+)HLM
5,6-DPQ	20.24	0.07094
6OHPQQI	24.84	0.5229
PQQI	54.25	8.552
PQ	53.69	20.15
5-HPQ	80.86	28.41

Supplementary Table 3

Comparison of anti-gametocyte activity without and after CYP2D6 conversion.

Table Analyzed	5-HPQ 2D6	PQQI 2D6	5,6-DPQ 2D6	6OHPQQI 2D6	PQ 2D6
Column A	(-)2D6	(-)2D6	(-)2D6	(-)2D6	(-)2D6
vs	vs	vs	vs	vs	vs
Column B	(+)2D6	(+)2D6	(+)2D6	(+)2D6	(+)2D6
Mann Whitney test					
P value	0.0079	0.0079	0.0159	0.0159	0.0079
Exact or approximate P value?	Exact	Exact	Exact	Exact	Exact
P value summary	**	**	*	*	**
Are medians signif. different? (P < 0.05)	Yes	Yes	Yes	Yes	Yes
One- or two-tailed P value?	Two-tailed	Two-tailed	Two-tailed	Two-tailed	Two-tailed
Sum of ranks in column A,B	40 , 15	40 , 15	39 , 16	39 , 16	40 , 15
Mann-Whitney U	0	0	1	1	0

Supplementary Table 4

Comparison of anti-gametocyte activity after CYP2D6 conversion in the presence or absence of paroxetine.

Table Analyzed	5-HPQ Paroxetine	PQQI Paroxetine	5,6-DPQ Paroxetine	6OHPQQI Paroxetine	PQ Paroxetine
Column A	(+)2D6	(+)2D6	(+)2D6	(+)2D6	(+)2D6
vs	vs	vs	vs	vs	vs
Column B	(+)2D6+Paroxetine	(+)2D6+Paroxetine	(+)2D6+Paroxetine	(+)2D6+Paroxetine	(+)2D6+Paroxetine
Mann Whitney test					
P value	0.1905	0.4127	0.1905	0.1905	0.0159
Exact or approximate P value?	Exact	Exact	Exact	Exact	Exact
P value summary	ns	ns	ns	ns	*
Are medians signif. different? (P < 0.05)	No	No	No	No	Yes
One- or two-tailed P value?	Two-tailed	Two-tailed	Two-tailed	Two-tailed	Two-tailed
Sum of ranks in column A,B	19 , 26	21 , 24	19 , 26	19 , 26	15 , 30
Mann-Whitney U	4	6	4	4	0

Supplementary Table 5

Steady-state kinetic parameters of human cytochrome NADH:P450 reductase (CPR) for primaquine and primaquine metabolites.

Compound	K_m (μM)	K_{cat} (min^{-1})	K_{cat}/K_m ($min^{-1}μM^{-1}$)
5-HPQ	61.56 ± 9.652	1,458±80	24.019±1.254
PQQI	55.932±9.776	1,498.5±107.075	27.159±1.469
5,6-DPQ	19.624±1.437	1,490.6±74.11	76.218±2.508
6OHPQQI	51.397±18.93	1,402±204.682	29.245±3.555
PQ[a]	1,655.5±871.791	1,574±465.695	1.041±0.118

[a] The high PQ concentration needed to reach V_{max} interfered with absorbance readings, therefore estimates from Michaelis–Menten fitted equation are shown.

Supplementary Table 6

Comparison of anti-gametocyte activity without and after huCPR conversion.

	5-HPQ	PQQI	5,6-DPQ	6OHPQQI
Table Analyzed	huCPR	huCPR	huCPR	huCPR
Column A	NoCPR	NoCPR	NoCPR	NoCPR
vs	vs	vs	vs	vs
Column B	huCPR	huCPR	huCPR	huCPR
Mann Whitney test				
P value	0.0002	0.0002	0.0002	0.0002
Exact or approximate P value?	Exact	Exact	Exact	Exact
P value summary	***	***	***	***
Are medians signif. different? (P < 0.05)	Yes	Yes	Yes	Yes
One- or two-tailed P value?	Two-tailed	Two-tailed	Two-tailed	Two-tailed
Sum of ranks in column A,B	100 , 36	100 , 36	100 , 36	100 , 36
Mann-Whitney U	0	0	0	0

Supplementary Table 7

Comparison of anti-gametocyte activity after huCPR conversion in the presence or absence of Pyruvate.

Table Analyzed	5-HPQ Pyruvate	PQQI Pyruvate	5,6-DPQ Pyruvate	6OHPQQI Pyruvate
Column A	huCPR	huCPR	huCPR	huCPR
vs	vs	vs	vs	vs
Column B	huCPR+Pyruvate	huCPR+Pyruvate	huCPR+Pyruvate	huCPR+Pyruvate
Mann Whitney test				
P value	0.004	0.004	0.004	0.004
Exact or approximate P value?	Exact	Exact	Exact	Exact
P value summary	**	**	**	**
Are medians signif. different? (P < 0.05)	Yes	Yes	Yes	Yes
One- or two-tailed P value?	Two-tailed	Two-tailed	Two-tailed	Two-tailed
Sum of ranks in column A,B	36 , 42	36 , 42	36 , 42	36 , 42
Mann-Whitney U	0	0	0	0
